# Kangaroo mother care: a multi-country analysis of health system bottlenecks and potential solutions

**DOI:** 10.1186/1471-2393-15-S2-S5

**Published:** 2015-09-11

**Authors:** Linda Vesel, Anne-Marie Bergh, Kate J Kerber, Bina Valsangkar, Goldy Mazia, Sarah G Moxon, Hannah Blencowe, Gary L Darmstadt, Joseph de Graft Johnson, Kim E Dickson, Juan Gabriel Ruiz Peláez, Severin Ritter von Xylander, Joy E Lawn, On behalf of the KMC Research Acceleration Group

**Affiliations:** 1 Innovations for Maternal, Newborn and Child Health, Concern Worldwide US, 355 Lexington Avenue, New York, NY 10017, USA; 2 Health Section, Programme Division, UNICEF Headquarters, 3 United Nations Plaza, New York, NY 10017, USA; 3 MRC Unit for Maternal and Infant Health Care Strategies, University of Pretoria, Private Bag X323, Arcadia 0007, Pretoria, South Africa; 4 Saving Newborn Lives, Save the Children, 2000 L Street NW, Suite 500, Washington, DC 20036, USA; 5 USAID's Maternal and Child Survival Program, 455 Massachusetts Avenue NW, Suite 1000, Washington, DC 20001, USA; 6 Maternal, Adolescent, Reproductive and Child Health (MARCH) Centre, London School of Hygiene and Tropical Medicine, London, WC1E 7HT, UK; 7 Department of Infectious Disease Epidemiology, London School of Hygiene and Tropical Medicine, London, WC1E 7HT, UK; 8 Department of Pediatrics, Stanford University School of Medicine, Stanford, CA 94305, USA; 9 School of Medicine, Pontificia Universidad Javeriana, Carrera 7 No 40-62, Bogotá, Colombia; 10 Fundación Canguro, Calle 56A No 50-36 - Bloque A13, Apto 416, Pablo VI Azul, Bogotá, Colombia; 11 Hospital Universitario San Ignacio, Carrera 7 No 40-62, Bogotá, Colombia; 12 Department of Maternal, Newborn, Child and Adolescent Health, World Health Organization, 20 Avenue Appia, 1211 Geneva 27, Switzerland

**Keywords:** Neonatal, newborn, preterm, kangaroo mother care, skin-to-skin care, health systems, mortality, quality

## Abstract

**Background:**

Preterm birth is now the leading cause of under-five child deaths worldwide with one million direct deaths plus approximately another million where preterm is a risk factor for neonatal deaths due to other causes. There is strong evidence that kangaroo mother care (KMC) reduces mortality among babies with birth weight <2000 g (mostly preterm). KMC involves continuous skin-to-skin contact, breastfeeding support, and promotion of early hospital discharge with follow-up. The World Health Organization has endorsed KMC for stabilised newborns in health facilities in both high-income and low-resource settings. The objectives of this paper are to: (1) use a 12-country analysis to explore health system bottlenecks affecting the scale-up of KMC; (2) propose solutions to the most significant bottlenecks; and (3) outline priority actions for scale-up.

**Methods:**

The bottleneck analysis tool was applied in 12 countries in Africa and Asia as part of the *Every Newborn *Action Plan process. Country workshops involved technical experts to complete the survey tool, which is designed to synthesise and grade health system "bottlenecks", factors that hinder the scale-up, of maternal-newborn intervention packages. We used quantitative and qualitative methods to analyse the bottleneck data, combined with literature review, to present priority bottlenecks and actions relevant to different health system building blocks for KMC.

**Results:**

Marked differences were found in the perceived severity of health system bottlenecks between Asian and African countries, with the former reporting more significant or very major bottlenecks for KMC with respect to all the health system building blocks. Community ownership and health financing bottlenecks were significant or very major bottlenecks for KMC in both low and high mortality contexts, particularly in South Asia. Significant bottlenecks were also reported for leadership and governance and health workforce building blocks.

**Conclusions:**

There are at least a dozen countries worldwide with national KMC programmes, and we identify three pathways to scale: (1) champion-led; (2) project-initiated; and (3) health systems designed. The combination of all three pathways may lead to more rapid scale-up. KMC has the potential to save lives, and change the face of facility-based newborn care, whilst empowering women to care for their preterm newborns.

## Background

### Vulnerability of preterm and/or small for gestational age newborns

Globally, 2.8 million newborns die each year, comprising 44% of under-five child deaths [1]. Newborns in low- and middle-income countries (LMICs) contribute to 98% of this burden, with more than three-quarters of the deaths in sub-Saharan Africa and South Asia - the very regions where progress for saving newborn lives is slowest [2]. Only recently has newborn health begun to emerge as a global and national public health priority, especially through attention to child survival in the Millennium Development Goals [3], and the *Born Too Soon *and *Every Newborn *[4] movements designed to specifically accelerate action for newborns.

Preterm birth accounts for an estimated 3.1% of all global disability-adjusted life years, directly through 1.1 million deaths and indirectly as a risk factor for many other cause-specific newborn deaths [5,6]. Each year, there are an estimated 15 million preterm newborns (born before 37 weeks of gestation) [7], most of whom are low birth weight (LBW) (<2500 g) [2]. The commonest underlying causes of LBW are prematurity, intrauterine growth restriction, or a combination of the two [2]. Africa has the highest rates of preterm birth [7] and South Asia has the highest rates of intrauterine growth restriction [2]. The time immediately after birth presents the greatest risk of death, which is exacerbated for preterm newborns as they have less physiological reserve, greater challenges with temperature regulation, immature organs (especially lungs, leading to respiratory distress syndrome), poor immune function, and heightened vulnerability to severe infections putting them at risk for problems associated with the transition to extra-uterine life [8,9]. The outcome of a preterm baby is a sensitive test of health system function; the highest preterm-specific mortality is in Sierra Leone [2], where health system gaps have now been illuminated by Ebola but have always been present.

### Kangaroo mother care definition

KMC is an approach to the care of preterm and/or LBW infants, which engages and empowers mothers and families as the main providers of the biological (warmth and food) and psycho-emotional (contact, caring, bonding and comfort) needs of their newborn. The cornerstone of KMC is the kangaroo position whereby the infant is placed and held in direct skin-to-skin contact on the mother's chest in an up-right position under her clothes. The aim is for early initiation of KMC and for continuous performance (over 18 hours per day), but initiation, continuity and duration may vary according to the stability of the infant and the context of care. Other key components of KMC are support for exclusive and early breastmilk provision and timely discharge from the hospital with appropriate follow-up [10]. KMC was first developed and scientifically evaluated in Colombia over three decades ago as an alternative to incubator care [10-13]. The evidence generated in Colombia allowed authorities to gradually include KMC in national LBW guidelines and spread the practice to a large number of health facilities. Recently, with preterm birth becoming the leading cause of under-five mortality, and additional evidence on KMC's mortality benefit, more attention has been focused on scaling up the practice.

Meta-analyses show that KMC reduces neonatal mortality, halving deaths among LBW babies weighing <2000 g [14,15]. KMC has multiple other benefits, including reductions in infection and sepsis by nearly 60% [2,15,16], as well as reductions in hypothermia and lower respiratory tract disease, and improved duration of exclusive breastfeeding, weight gain, length and head circumference, maternal-infant bonding and long-term child development and health [10,14-21]. Intermittent KMC may also be beneficial, especially to non-mortality outcomes (higher rates of breastfeeding, better short-term physiological regulation, maternal bonding, amongst others), but as yet there are limited data.

**Figure 1:**
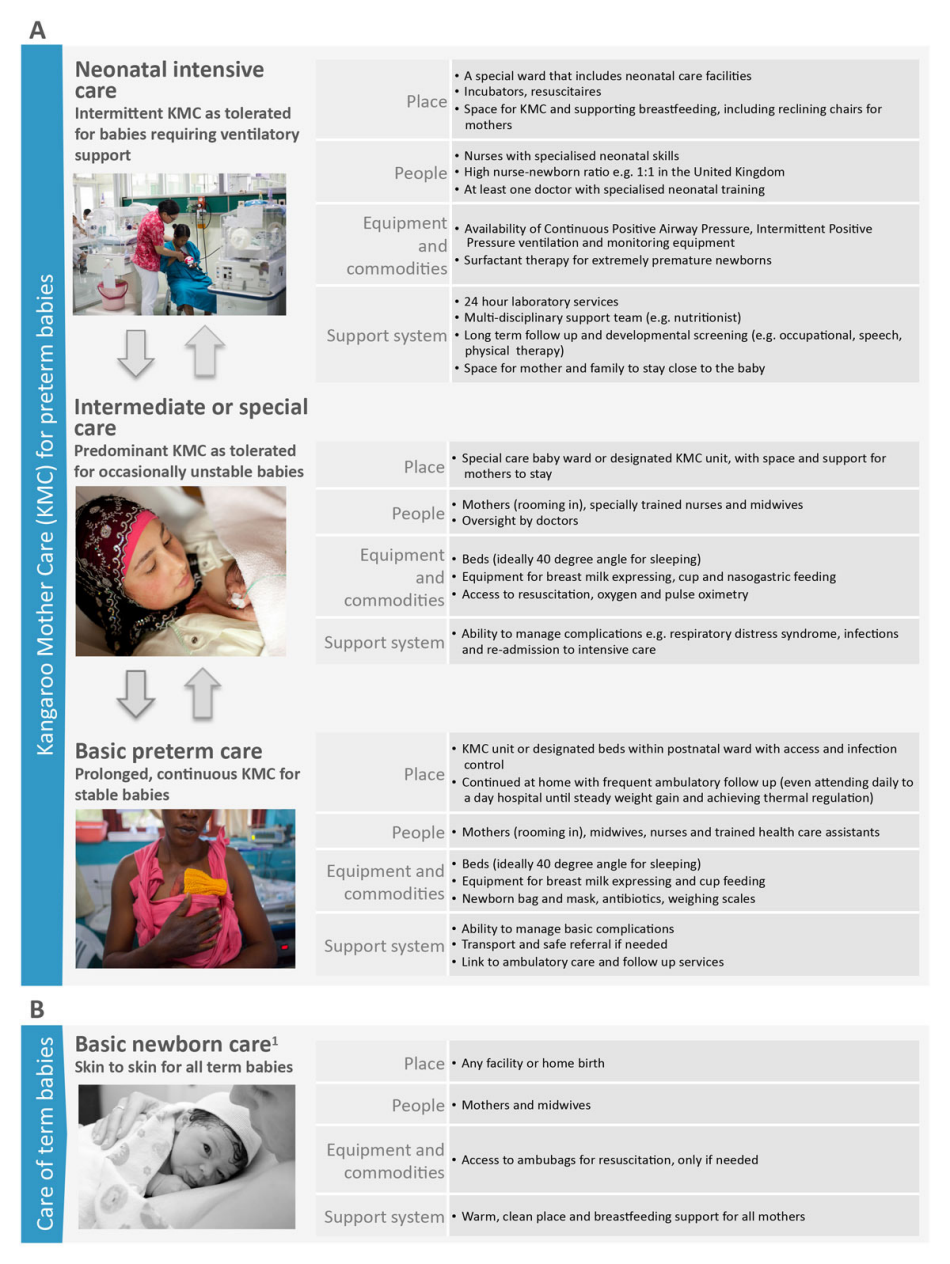
**Kangaroo Mother Care, showing health systems requirements by level of care**. Any items available at the basic level should be available at the higher level. For more details of special care and neonatal intensive care requirements see Moxon *et al*. paper on inpatient care of small and sick newborns in this supplement. KMC: Kangaroo Mother Care. Part A: Kangaroo mother care for preterm babies. Part B: Care of term babies. ^1^KMC is not the same as skin-to-skin care alone. KMC involves continuous prolonged skin-to-skin contact with the infant placed on top of the mother's chest in a prone vertical position (Kangaroo Position), support for breastmilk feeding and a supportive environment. Neonatal intensive care image source: Syane Luntungan/Jhpiego. Intermediate or special care image source: ^©^EFCNI. Basic preterm care image source: Save the Children. Basic newborn care image source: Joyce Godwin.

KMC has been formally endorsed by the World Health Organization (WHO) for stabilised babies <2000 g in health facilities as a safe complement to conventional neonatal care [22,23]. The evidence to date is for facility implementation of KMC and continuation at home post-discharge, and as yet community-based initiation of KMC is not recommended. Figure 1 shows how skin-to-skin care and KMC can be integrated within the health system, but this may look different in different health system contexts and levels of care. KMC is a cornerstone of facility-based care for small and sick babies, and can complement neonatal intensive care of extremely premature babies. KMC is embedded in the broader continuum of care for small and sick newborns, including obstetric care [24], management of preterm labour [25], basic newborn care and resuscitation [26], management of infections [27] and more comprehensive care of small and sick newborns, especially those with respiratory complications [28].

The distinction between KMC and skin-to-skin care should be clear. Skin-to-skin care for term newborns - where babies are placed on their mothers chest directly after birth as part of basic newborn care [26] and intermittently thereafter - helps to promote warmth, bonding and breastfeeding as part of a continuum of woman and baby-centred care. KMC is intended for infants <2000 g with the aim of thermal regulation achieved through continuous skin-to-skin contact in the KMC position. KMC may be required for weeks and is carried out alongside other aspects of care for the preterm baby.

Now, 44% of the 75 Countdown to 2015 countries report that they have a national policy on KMC in facilities for LBW/preterm newborns [3]. In many cases, these policies are recent and implementation is limited. Countries may initiate KMC and fail to increase coverage [29]. Unfortunately, coverage data are not available as yet, but are urgently needed. Hence, in-depth country analyses and case studies remain critical to learning what works and what does not.

The objectives of this paper are:

1. To use a 12-country analysis to explore health system bottlenecks affecting the scale-up of KMC.

2. To present proposed solutions to overcome the most significant bottlenecks for KMC based on learning from the 12-country analysis, a literature review and programme experience.

3. To discuss policy and programmatic implications and propose priority actions for KMC scale-up.

## Methods

This study used quantitative and qualitative research methods to collect and assess health system bottlenecks and solutions to the scale-up of maternal and newborn care interventions in 12 countries: Afghanistan, Bangladesh, Cameroon, Democratic Republic of Congo (DRC), India, Kenya, Malawi, Nigeria, Nepal, Pakistan, Uganda, and Vietnam.

### Data collection

As part of the development of the *Every Newborn *Action Plan (ENAP), the bottleneck analysis tool was developed to assist countries in identifying context-specific bottlenecks to the scale-up and provision of maternal and newborn health interventions across the seven WHO health system building blocks (see Additional file 1) [30,31]. The tool was applied during a series of national consultations between July 1^st ^and December 31^st^, 2013. The workshops for each country included participants from national ministries of health, United Nations agencies, the private sector, non-governmental organisations, professional bodies, academia, bilateral agencies and other institutions. For each workshop, a facilitator, oriented on the tool, coordinated the process and guided groups to reach consensus on the specific bottlenecks for each health system building block. This paper, fifth in the series, focuses on KMC.

In the tool, KMC was defined as a package with two main behaviours selected as tracers: (1) continuous skin-to-skin care, placing and securing (usually with a cloth) a baby on a mother or other caregiver's bare chest; and (2) frequent and exclusive breastfeeding including support for small and sick babies who cannot feed directly from the breast.

### Data analysis methods

**Figure 2:**
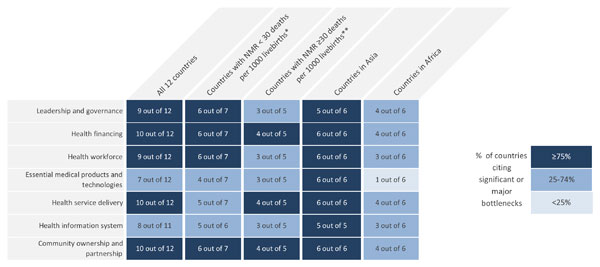
**Very major or significant health system bottlenecks for kangaroo mother care**. NMR: Neonatal mortality rate. *Cameroon, Kenya, Malawi, Uganda, Bangladesh, Nepal, Vietnam. **Democratic Republic of Congo, Nigeria, Afghanistan, India, Pakistan. See additional file 2 for more details.

**Figure 3:**
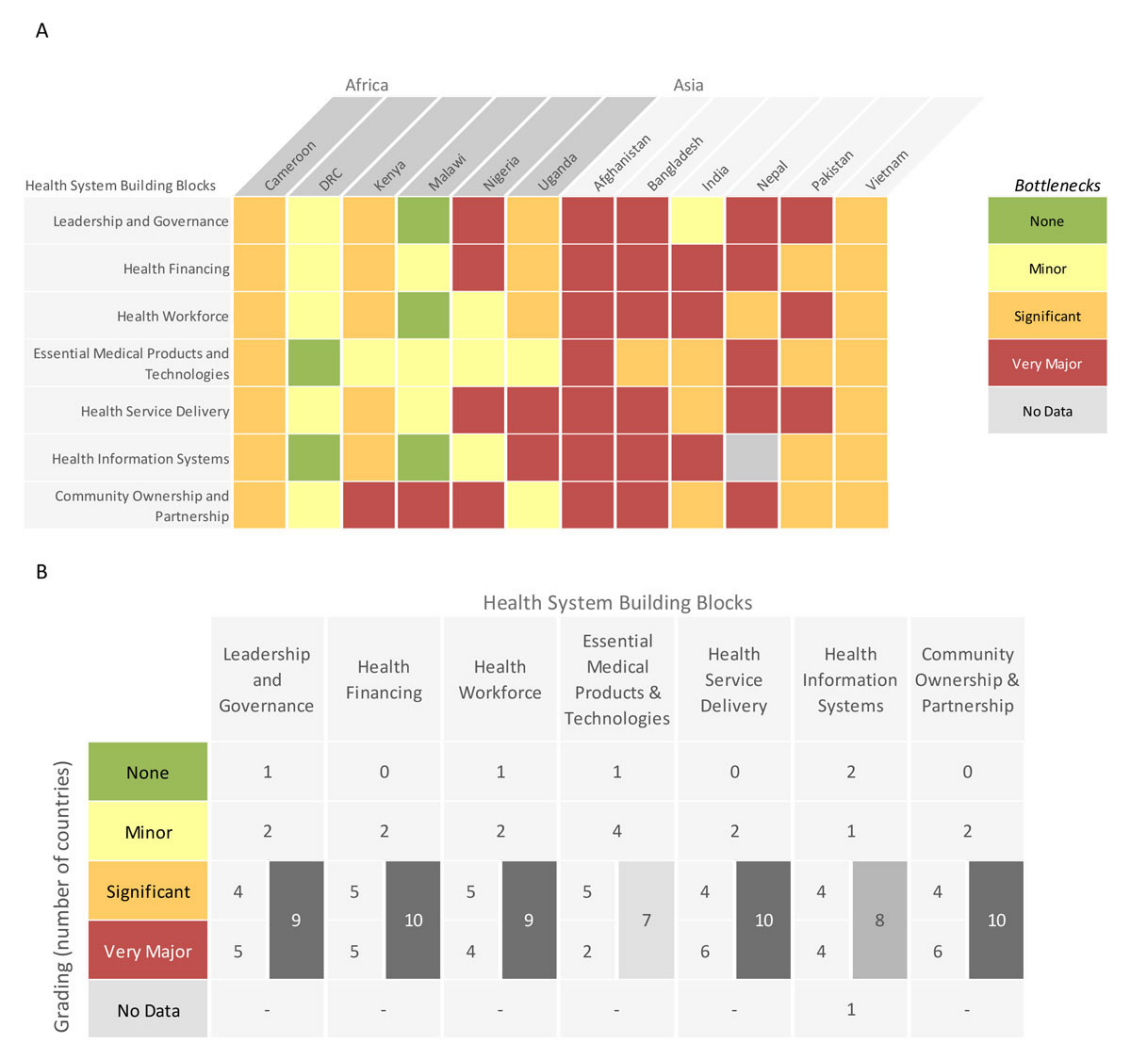
**Individual country grading of health system bottlenecks for kangaroo mother care**. Part A: Heat map showing individual country grading of health system bottlenecks for kangaroo mother care. Part B: Table showing total number of countries grading significant or major for calculating priority building blocks. DRC: Democratic Republic of the Congo.

We graded country-specific bottlenecks for each health system building block using one of the following options, which were derived from the bottleneck analysis tool that was completed by country teams: not a bottleneck (=1), minor bottleneck (=2), significant bottleneck (=3), or **very major **bottleneck (=4). We first present the grading in heat maps according to the very major or significant health system bottlenecks as reported by all 12 countries, then by mortality contexts (neonatal mortality rate [NMR] <30 deaths per 1000 live births and NMR ≥30 deaths per 1000 live births) and then by region (countries in Africa and countries in Asia) (Figure 2). We developed a second heat map showing the specific grading of bottlenecks for each health system building block by individual country (Figure 3). Finally, we categorised context-specific solutions to overcome challenges to scaling up KMC identified in all countries into thematic areas linked to the specific bottlenecks (Table 1 and Table S2, additional file 2). We undertook a literature review to identify further case studies and evidence-based solutions for each defined thematic area (see Additional file 2).

**Table 1:** Summary of bottlenecks and solution themes for scale-up of kangaroo mother care.

Health system building block	Subcategory	Significant bottleneck	Number of countries	Solution themes
Leadership & governance	Policy and guidelines	Absence of national KMC policies/strategies and/or creation and dissemination of service guidelines to support the implementation and scale-up of KMC	10	• Advocacy and sensitisation • Policies / include in national MCH objectives / guidelines/ strengthen role of Ministry of Health
	Awareness	Poor or no awareness of KMC by leadership	3	• Implementation modalities and curriculums

Health financing	Funding	Funding limited or not available for implementation and scale-up	6	• Advocacy for increased budget/funding
	Out-of-pocket costs	Burden of out-of-pocket expenditures by caregivers	6	• Development of a costed master plan
	Policy	Lack of integration of KMC in national costing plans/policies	2	• Increased donor support • Insurance and/or community financing schemes

Health workforce	Number, competence, distribution of health workers	Shortage of competent health workers and poor distribution of properly trained personnel authorised to provide care for LBW babies and support KMC	8	• Training and capacity development
	Training	Lack of training of health workers on KMC	7	• Increasing number and capacity of health workers and creating a dedicated cadre for KMC
	Mentorship and supervision	Lack of mentorship and supervision mechanisms for KMC	5	• Development and implementation of job descriptions and mentoring guidelines
	Knowledge and awareness	Poor knowledge and awareness of health workers regarding importance and utility of KMC	4	• Setting up supervision and monitoring and evaluation mechanisms
	Attitudes	Negative health worker attitudes towards KMC	3	
	Job descriptions	Lack of job descriptions for health workers supporting KMC	3	

Essential medical products & technologies	Resources	Unavailability of resources and supplies needed to perform KMC	10	• Budget with funding for equipment
	Procurement	Poor procurement and supply chain logistics for KMC	2	• Procurement of basic supplies • Standard list of equipment in facilities

Health service delivery	Physical and logistical constraints	Lack of space and logistical constraints related to support and monitoring of mothers/caregivers performing KMC	8	• Investment in space
	Quality	Poor quality of care issues and lack of quality improvement related to the implementation of KMC	4	• KMC follow-up made a part of existing services
	Follow-up	Lack of follow-up of KMC practice after discharge	3	• Integration of KMC and breastfeeding promotion
	Availability and delivery	Unavailability of services / disparities in delivery of KMC	2	
	Referral system	Lack of referral system in place for KMC (transport and access)	1	

Health information system	Availability of information	Lack of information, records and data on coverage of KMC and non-use of information when available	11	• Development of indicators and inclusion in records
	Quality of information	Poor quality information available on LBW babies	2	• Capacity building for use of data to monitor trends and improve services

	Knowledge and awareness	Lack of awareness and knowledge of and mobilisation around KMC in the community	10	• Increase awareness including amongst men
	Promotion	Lack of proper mechanisms to promote KMC in the community	5	• Promotion of KMC in the community (better IEC programmes and empowerment of community health workers in KMC; translation of material to local languages)
Community ownership & partnership	Socio-cultural barriers	Socio-cultural barriers to the practice of KMC	4	• Community empowerment and acceptance (action plan to address community perception)
	Acceptability	Lack of acceptability of KMC among community members	3	
	Engagement	Poor or no engagement and support of men and the community	2	
	Financial barriers	Financial barriers to support KMC uptake at the community level	1	
	Access	Poor access to services at the health facility	1	

For more information on methods, detailed analysis of the steps taken to analyse the intervention-specific bottlenecks and limitations, please refer to the overview paper [31].

## Results

National level responses to the bottleneck analysis tool were analysed for 10 countries (Afghanistan, Bangladesh, Cameroon, Democratic Republic of Congo, Kenya, Malawi, Nigeria, Nepal, Uganda, and Vietnam). India and Pakistan undertook sub-national data collection for two states and five provinces, respectively [30]. All of the countries/subnational regions completed the KMC section regarding bottlenecks, solutions and grading with the exception of Nepal, which did not grade for the health information systems building block. Nepal was therefore removed from the sample for the quantitative grading of this building block, but included in the analysis of all the other building blocks.

Grading according to the number of countries that reported significant or very major health system bottlenecks for the scale-up of KMC is shown in Figure 2. Most countries reported significant or very major bottlenecks for health financing (10 countries), community ownership and partnership (10 countries), and health service delivery (10 countries). Health financing, and community ownership and partnership bottlenecks were significant or very major across countries overall, in both low and high mortality contexts and in the Asia region; this suggests that these two building blocks may be priorities to tackle in order to promote the scale-up of KMC.

Figure 3 breaks down the grading of health system building blocks reported for each individual country. Regional differences were reported in the perceived severity of health system bottlenecks, and, thus, the perceived feasibility of KMC scale-up. Asian countries reported very major or significant bottlenecks for all the building blocks except for one country that did not consider leadership and governance as a bottleneck. The difference between mortality contexts was less marked (Figure 2).

Significant bottlenecks also existed across other health system building blocks, particularly leadership and governance (9 countries) and health workforce (9 countries) (Figures 2 and 3).

Table 1 summarises the main bottlenecks and general solution themes identified by countries for each of the seven health system building blocks, which are described below.

## Leadership and governance bottlenecks and solutions

Leadership and governance was rated as having significant or very major bottlenecks across nine out of 12 countries (Figure 2). Ten out of 12 countries highlighted the absence of a national KMC policy and/or absence of the existence and dissemination of KMC service guidelines, even in facilities already promoting the practice (Table 1). Complete absence of policy and guidelines was more common in Asian countries while poor dissemination of guidelines was more common in African counties. Workshop participants reported an overall lack of prioritisation of KMC by regulatory bodies and lack of institutionalisation of KMC.

Proposed solutions to address bottlenecks centred on advocacy to increase awareness, budget and resources for KMC; strengthening the role of the ministries of health; and sensitisation of the community and health workers to the benefits of KMC.

### Health financing bottlenecks and solutions

Health financing was rated as having significant or very major bottlenecks across 10 out of 12 countries (Figure 2). Lack of finances for health at district and national levels and out-of-pocket expenses (the specific connection with KMC was not noted) were each reported as the main obstacles to the implementation and scale-up of KMC by six country teams (Table 1).

Proposed solutions included advocacy for increased donor support and budget for maternal and newborn health and evaluation; the development of costed master plans to support dissemination and the use of policy and guidelines; and the introduction of health finance protection schemes.

### Health workforce bottlenecks and solutions

Health workforce was rated as having significant or very major bottlenecks across nine out of 12 countries: all six countries in Asia and three of six countries in Africa (Figure 2). Eight out of 12 country teams reported specific health workforce gaps affecting the uptake of KMC, including the shortage and poor distribution of well-trained health workers to care for LBW babies and support the practice of KMC (Table 1). Additional bottlenecks included lack of adequate mentorship and supervision mechanisms, and knowledge and awareness of health workers.

The most commonly proposed solution centred on improvements in training and capacity development and the creation of national newborn health training curricula that include KMC as a priority intervention. The development and implementation of job descriptions, mentoring guidelines, and supervision, monitoring and evaluation mechanisms were also mentioned.

### Essential medical products and technologies bottlenecks and solutions

The essential medical products and technologies building block was rated as having significant or very major bottlenecks across seven out of 12 countries: all six countries in Asia and only one of six countries in Africa (Figure 2). Ten countries reported poor availability of basic supplies for KMC in health facilities, particularly those required to support the feeding of LBW babies (Table 1). Procurement and supply chain issues were also mentioned.

Proposed solutions included developing a standard list and budget for equipment needed to promote KMC and procuring basic supplies.

### Health service delivery bottlenecks and solutions

Health service delivery was rated as having significant or very major bottlenecks across 10 out of 12 countries (Figure 2). Eight countries reported that facilities did not have adequate space for the performance and monitoring of KMC. Presence of poor referral and transport systems, poor quality of KMC delivery and weak quality improvement measures were also reported (Table 1).

Solutions included conducting advocacy and creating policies to encourage investment in space for KMC and making KMC follow-up a part of existing postnatal services. Additionally, participants from Vietnam proposed a quality improvement solution, which involved integrating KMC and breastfeeding promotion into a scoring system for evaluation of health facility performance.

### Health information system bottlenecks and solutions

The health information system building block was rated as having significant or very major bottlenecks across eight out of 11 countries (Nepal did not do grading), all five countries in Asia and three of six in Africa (Figure 2). It was widely recognised (11 out of 12 countries) that scale-up of KMC was hindered by the lack of coverage data in the existing health information system (Table 1). Poor quality of data on LBW and preterm birth was also mentioned.

The main proposed solutions were to improve KMC metrics by defining standard KMC indicators and incorporating them in routine data collection tools and platforms; and putting in place better reporting and clinical audits in KMC units.

### Community ownership and partnership bottlenecks and solutions

Community ownership and partnership was rated as having significant or very major bottlenecks in 10 out of 12 countries (Figure 2). Ten of 12 country teams reported a lack of awareness, education and community mobilisation to increase knowledge about the benefits of KMC (Table 1). Apart from the grading, there were some differences in the specific bottlenecks between Africa and Asia. In Africa, country issues included large distances to health facilities (1 country), lack of data on acceptability of KMC in the community (1 country), existing customs of carrying babies on the back (1 country), and misconceptions including the belief that the most effective care is in an incubator (1 country). Asian countries reported that this was a new technique (1 country) and the perception that KMC was both not feasible in hot, humid environments and that privacy was a concern in its implementation (1 country). Country teams also mentioned the lack of information, education and communication materials in the local language and poor involvement and support of men and the general community.

Solutions proposed included better health promotion programmes, empowerment of community health workers in KMC and development of an action plan to address community perceptions.

## Discussion

With increased attention and investment in the newborn through the ENAP [4], the *Born Too Soon *report [10] and a recent call for acceleration of KMC [32], KMC has been highlighted and promoted as a high impact intervention that can save lives when implemented at scale. Many countries are taking up KMC, with 33 of 55 priority countries reporting that they have a national policy for KMC in facilities for LBW/preterm babies [3]. To date, there has been limited progress for KMC implementation. Hence, there was an immediate need for a systematic, multi-country analysis of bottlenecks and solutions, and learning from countries that are further along the path to scale. The first global analysis of the barriers to KMC implementation took place in 1998 at the second international KMC workshop held in Bogota [33]; participants highlighted the need for policies, advocacy, dissemination and financial investment linked to political will. Many of these factors, as demonstrated through our findings remain important priorities. The *Every Newborn *Lancet Series highlighted the need for prompt and deliberate prioritisation of KMC as part of the management of small babies. Our findings highlight, support and further explore the conclusions made by Dickson and colleagues regarding regional differences between Africa and Asia for perceived challenges to the scale-up of KMC [30]. These differences are further discussed below considering country pathways to scale and examples of regional networks for implementation.

Fewer countries reported constraints with products and technologies and the health information system, reflecting the strength of KMC as a person-driven intervention. The most significant or very major bottlenecks were reported for: health financing (10 countries), community ownership and partnership (10 countries), health service delivery (10 countries), leadership and governance (9 countries) and health workforce (9 countries). Health financing and community ownership and partnership bottlenecks were significant or very major overall, in both low and high mortality contexts and in the Asia region (Figures 2, 3a and 3b). We will discuss each of these in turn.

### Health financing priority actions

Financial barriers are commonly experienced in various sectors in LMICs, but newborns have been particularly neglected with respect to budgeting, and allocation of national funds and donor investments [28,31,34]. The main reported health financing bottlenecks were related to lack of policies and integration of budgets into national plans and limited funding, which are heavily influenced by leadership and governance. Additionally, concerns were expressed around the burden placed on families by out-of-pocket costs which is a general maternal, newborn and child health (MNCH) financing concern not limited to KMC or newborns, although newborns may have been particularly missed in insurance schemes and financial protection mechanisms [28,31]. Addressing health financing gaps in MNCH care will help to tackle health financing barriers reported by country teams for the scale-up of KMC. This necessitates government oversight to advocate for, track and govern the allocation of funding for MNCH interventions, with a commitment to integrate KMC into national strategies, implementation guidelines and operational management. Payment schemes (e.g. community-based insurance and mutual health) are needed to lessen the burden of out-of-pocket expenses for families [28,30,35]. Public awareness is important to advocate for alternative financing mechanisms to address financial bottlenecks for the underserved [30].

A costed health sector plan for reproductive, maternal, newborn and child health should include KMC as part of newborn care, with a clearly linked implementation strategy adapted at national and sub-national levels. Cost-effectiveness analyses are helpful in promoting KMC and addressing bottlenecks to its uptake; for example, those related to health financing (e.g. direct costs for families and cost savings for health facilities), health service delivery (e.g. duration of hospital stay) and health workforce (e.g. staff load) [36-38]. Cost-effectiveness analyses have been undertaken in Nicaragua [39], Ethiopia [40,41] and Colombia [38] showing the cost savings of KMC versus standard care. In Nicaragua, KMC was found to be less expensive than standard care even without including the long-term health and economic benefits of improved cognitive abilities and reduced stunting and wasting. In Colombia, the Kangaroo Foundation developed a package for public and private insurance covering all of the costs of care for a KMC infant including follow-up for one year of corrected age; this could be useful in defining a minimal universal KMC package (Personal communication with Nathalie Charpak).

The bottlenecks around health financing are further exposed by the fact that a pathway for KMC scale-up in low-income countries, like for many other interventions, has been donor initiated and has resulted in countries being dependent on this funding. Including KMC within the Global Financing Fund for Reproductive, Maternal, Newborn and Child Health by the World Bank could be a welcome shift to a systems approach to scaling up and institutionalising KMC [42]. This donor-led entry point has been translated to institutionalisation and health sector funding in a number of countries such as Tanzania and Rwanda.

### Community ownership and partnership priority actions

**Figure 4:**
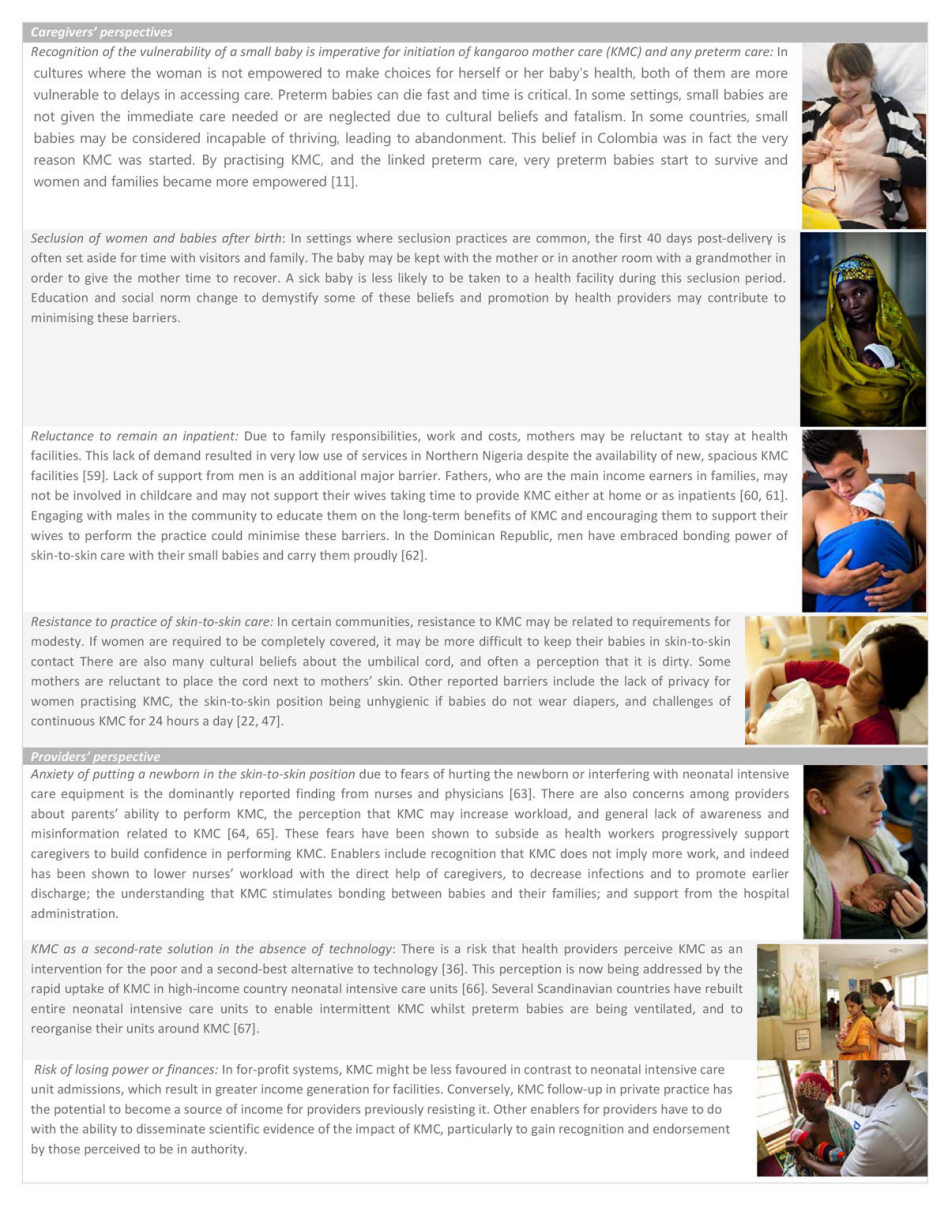
**Overcoming socio-cultural barriers to the scale-up of kangaroo mother care and preterm care**. Both caregivers and providers may have barriers to the uptake of KMC. Local context must be taken into account to understand and overcome these barriers. Based on literature and programme experience of the authorship team, we summarise some of the common barriers faced and enablers found. KMC: kangaroo mother care. Mother practicing KMC image source: Save the Children. Mother practicing KMC image source: Pep Bonet/NOOR for Save the Children. Father practicing KMC image source: Erica Pineros/Save the Children. Mother practicing KMC image source: ^©^EFCNI. Mother practicing KMC with baby with long-term oxygen treatment image source: ^©^Fundación Canguro. Mother practicing KMC with nurse by her side: Ritam Banerjee/Save the Children. Mother practicing KMC with twin babies: Jordi Matas/Save the Children

Respondents in the bottleneck workshops mentioned lack of community awareness of the burden of prematurity and the need for KMC as a major bottleneck. Evidence and international guidelines endorse KMC in health facilities and continuation at home post-discharge, but as yet the WHO does not recommend community-based initiation of KMC. Socio-cultural factors may hinder rapid and universal uptake of KMC, both by communities and health workers (Figure 4). Promotion of uptake of KMC involves engaging all of the community including cultural, religious, and community leaders, enlisting support from grandparents and family members, and shifting social norms around KMC positioning and skin-to-skin contact while avoiding stigmatisation of KMC provision as a failure to bear a full-term infant or to afford incubator care [35]. Workshop participants proposed formative research and a linked plan to address community perceptions that hinder the acceptance of KMC.

In Malawi, for example, there is now wide national awareness of KMC due to a national community sensitisation campaign through radio and community groups, and community change agents (e.g. grandparents) as well as distribution of family counselling materials promoting KMC [43-45]. This multi-channel promotion may have contributed to increased commitment to the implementation of KMC in facilities, demand by health providers and mothers, and ultimately uptake of KMC [46]. The bottleneck assessment participants also underlined the important role men could play in improving the uptake of KMC as a result of their traditional role as decision-makers. They could also physically support their partners by providing intermittent KMC. The latter has been seen in Latin American and Caribbean countries (Dominican Republic, Colombia and others) and in Europe, where fathers have become regular caregivers for preterm babies [47,48] (Figure 4).

Community health and extension workers, midwives and women experienced in practising KMC can promote it during antenatal care, home visits during pregnancy and in women's groups [35,49]. Where a large proportion of births take place at home, community health workers could facilitate identification of small babies. For example, in rural Tanzania and Uganda, a foot-length card has been used by community health workers to identify small babies, put them skin-to-skin and refer [50,51]. Promoting the benefit of skin-to-skin care for term babies at the community level could help to normalise KMC and advance community ownership.

**Figure 5:**
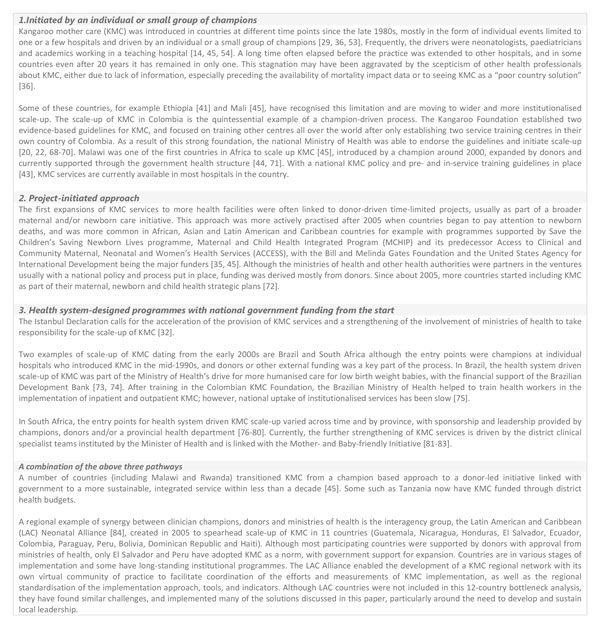
**Pathways from policy to implementation for kangaroo mother care**. Countries have followed different pathways in introducing and expanding implementation of KMC services. Based on a review of the processes in a small number of countries in Latin America, South Asia and Africa that now have KMC services in more than half of facilities that conduct births and adapting from previous work, we have identified three entry points: champion(s), a project-based approach, and a health system-designed programme [26,30]. KMC: kangaroo mother care. LAC: Latin American and Caribbean.

Although health worker support for KMC is imperative, KMC has the potential to change the health worker dominated model of care by empowering mothers and families to play a crucial role in their child's treatment plan [52]. KMC bottlenecks did not differ by mortality contexts, as for most other maternal and newborn interventions, but rather by region, which suggests that cultural perceptions may play a large role in the implementation of KMC. Community-led approaches may be more common in high-income settings where KMC is more frequently demanded by mothers and fathers (Figure 5). However, creating aspirations to adopt practices associated with high-income societies could potentially be utilised to stimulate greater demand in low-income settings.

### Health service delivery priority actions

KMC requires a continuum of care from facility to home and between the different levels of the health system [43]. Entry points and integration of KMC into existing services vary depending on the health systems context (Figure 1). In high mortality settings, the priority is saving lives with the efficient use of the limited availability of human and capital resources. In addition, KMC provides potential relief to busy midwifery and nursing staff, more rational use of space, and the potential for early discharge.

The goal of implementation of KMC or any other intervention is for the practice to become functional and integrated at all levels of the health system [53]. KMC services have been mainly introduced at tertiary facilities [14,29,45,54] despite most facility deliveries taking place at lower levels or at home. Wherever the majority of births take place, often at district level health facilities, space designated for KMC should be prioritised, close to the labour ward and adjacent to the neonatal intensive care unit or special care unit [55]. Country teams proposed tailoring health worker curricula and service guidelines to the level of care. Specific guidelines such as those for admission and discharge criteria, detailed ward protocols (e.g. rules about visitors and rooming-in, provision of meals and use of incubators) and relevant training for health providers are all essential for effective service delivery and for ensuring consistent quality of care at multiple sites in a given country.

As complexity of care has increased and as mortality has been reduced, middle-income countries, such as Colombia, Brazil, and South Africa have demonstrated the shift to using KMC as part of special care and neonatal intensive care. A number of high-income countries have recently placed major emphasis on KMC including substantial structural investments to integrate KMC into intensive care services (Figure 1). In many of these countries, the motivation is improved outcomes such as reduced disability, and family-centred care. In middle-income countries, there has been a gradual building of multi-disciplinary teams with centres of excellence for preterm baby care, including involvement of nutritionists, physiotherapists, speech therapists and other linked medical disciplines as well as more robust follow-up systems.

In low-income settings, compliance with referral of small babies to a health facility and providing ambulatory and follow-up services after facility discharge have been identified as critical challenges [45]. Solutions could include frequent and regular follow-up, at a peripheral lower-level site with appropriate outpatient care, or in the community via home visits to track growth and promote healthy care-taking behaviours [35], with referrals to higher level facilities to manage complications [45]. Preterm babies are especially vulnerable to weaknesses in referral systems.

### Leadership and governance priority actions

The bottleneck analysis results confirm the barriers related to the lack of awareness and commitment among leadership identified by Charpak and colleagues [47], which are essential for investment in a health system scale-up and especially for ongoing sustainability. Sensitising leadership at all levels (national and facility) to preterm birth care including KMC may positively influence more rapid scale-up.

The end goal of high coverage and quality of maternal and newborn care including KMC is the same, but the entry point and pathway to scale may vary [29]. The three main pathways for initiation of KMC that have been used in countries include: (1) initiation by an individual champion or small group; (2) project-based initiation; and/or (3) health system-designed programme (Figure 5). There are countries that have shifted from one to the next of these three approaches (e.g. Malawi) or have combined them (e.g. Rwanda).

### Health workforce priority actions

Limited information is available on human resources required to provide KMC services at different levels of health facilities and to promote skin-to-skin care in the community. Staff qualifications may vary according to the criteria set for the commencement of continuous KMC. Where stable babies start with continuous KMC at a lower weight or gestational age, highly qualified personnel should be available at all times. Where babies are enrolled into continuous KMC at a higher weight or gestational age or when mothers are practising intermittent KMC, task shifting may be possible, with lower-qualified but experienced staff providing support [55]. At one major hospital in Malawi, in the absence of qualified staff, patient attendants were trained to run the KMC ward under nursing and medical supervision [56]. Supervision by a senior professional nurse or other related cadre is crucial at all times, with limited or no staff rotation given the specialisation required to care for vulnerable LBW and preterm babies [55].

One important lesson learned is incorporation of KMC and other competency based maternal and newborn care into pre-service education and not relying on high cost, time-consuming, in-service training models.

### Limitations

General limitations of the bottleneck analysis tool and process, including its subjectivity, quality and length, are described in the first paper of this series [28]. The results are based on the knowledge and opinions of the individuals who completed the bottleneck analysis tool and may not capture all views and experiences in the country, particularly those of mothers and families.

The observed regional differences between Africa and Asia might not have been apparent had different countries been involved in the analysis. Some of the African countries (Cameroon, Malawi and Uganda) had a longer history of KMC implementation with more intentional scale-up support across the continent including inter-country learning visits, workshops and shared toolkits. Meanwhile in Asia some countries had a longer history (India and Vietnam), but less transferability and support across large, diverse settings [35]. However, the consistency of the grading between countries regarding the bottlenecks and the practical solutions proposed do provide valuable information for programmes.

### Future agenda

**Figure 6:**
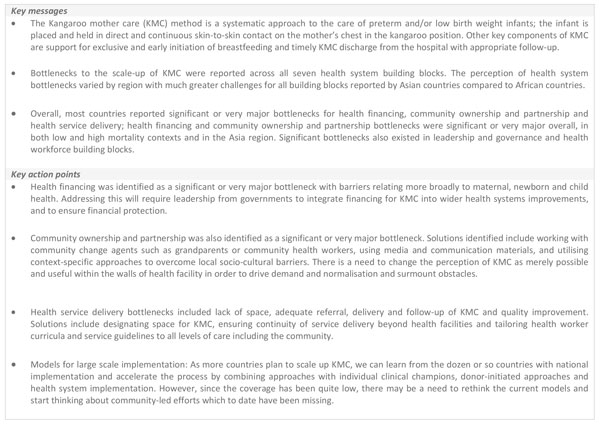
**Key messages and action points for scale-up of kangaroo mother care**. KMC: kangaroo mother care

Considering all the bottlenecks, experiences and strategies discussed above, a crucial step for most countries is to embed KMC in national health sector plans and define and disseminate a national KMC policy with specific service standards at each level, alongside rollout strategies that take into consideration the place of birth and needs of the community. Political commitment includes deriving and allocating adequate funds for scale-up. Finally, community awareness, mobilisation and overcoming socio-cultural barriers to normalise KMC are critical and require systematic, context-specific approaches. In doing so, bottlenecks to the other health system building blocks, particularly for data collection and monitoring and accountability, will need to be addressed (Figure 6). Validated indicators, especially for coverage, are a critical need and are being developed as part of the *Every Newborn *metrics work [57].

There are many research questions linked to this implementation agenda for KMC, particularly around context-specific solutions to the main challenges in terms of community and provider uptake, human resource innovations and novel financing strategies. Many of these are wider than KMC alone. In addition, improved costing data for programme planning will be key. Community initiation of KMC requires more rigorous testing but empowerment of women and communities and promotion of skin-to-skin care are also critical to widespread adoption of KMC.

## Conclusions

The global community is increasingly recognising the importance of saving newborn lives, and promoting a healthy start in life, particularly concentrated among babies born too soon or too small. The *Every Newborn *initiative is a response to national and global stakeholders' requests for coordinated, evidence-based action, and KMC is part of that plan [30,58]. Preventive interventions for preterm birth have limited impact at present. Other more complex therapeutic care for preterm babies is important but it will take more time and investment to develop equipped neonatal intensive care units and train staff to provide high-quality care. KMC can be gradually implemented as barriers are being addressed. Whilst a small number of countries in most regions (Latin America, Asia, Africa and Europe) have reached a measure of scale with KMC, many others are now designing how to integrate and scale up within their health system contexts. Given rapid on-going progress, this paper highlights the value of countries learning from one another, and highlights how to identify and overcome context-specific challenges so that every woman who has a preterm baby needing KMC and care will be able to provide this care.

## List of abbreviations

ENAP: Every Newborn Action Plan, KMC: Kangaroo Mother Care; LBW: Low Birth Weight; LMIC: Low and Middle Income Countries; MNCH: Maternal, Newborn and Child Health; NMR: Neonatal Mortality Rate; WHO: World Health Organization.

## Competing interests

The authors have not declared competing interests. The assessment of bottlenecks expressed during consultations reflects the perception of the technical experts and may not be national policy. The authors alone are responsible for the views expressed in this article and they do not necessarily represent the decisions, policy or views of the organisations listed, including WHO.

## Authors' contributions

LV carried out the analysis with detailed inputs from KED, JEL and SGM. LV wrote the manuscript. AB, KK, JEL, GD, BV, GM, and HB contributed to the drafting and critical revision of the manuscript. All authors read and commented on multiple drafts of the manuscript and approved the final manuscript.

## Supplementary Material

Additional file 1 **Format: PDF**. Bottleneck tool questionnaire. Click here for file

Additional file 2 **Format: PDF**. Supplementary tables, figures and literature search strategy. Click here for file

## Declarations

Work for this supplement was funded by the Bill and Melinda Gates Foundation through a grant to US Fund for UNICEF (Grant ID: OPP1094117), and support from Save the Children's Saving Newborn Lives Programme. Additional funding for the bottleneck analysis was received from USAID (Grant ID: GHA-G-00-07-00007) through UNICEF.

This article has been published as part of *BMC Pregnancy and Childbirth *Volume 15 Supplement 2, 2015: Every Woman, Every Newborn. The full contents of the supplement are available online at http://www.biomedcentral.com/bmcpregnancychildbirth/supplements/15/S2.
