# Supplementary material for: Kangaroo mother care: a multi-country analysis of health system bottlenecks and potential solutions
Source: BMC Pregnancy Childbirth. 2015 Sep 11;15(Suppl 2):S5. doi: 10.1186/1471-2393-15-S2-S5 (PMC4577801; doi:10.1186/1471-2393-15-S2-S5)
Supplement: Additional file 2 — Format: PDF. Supplementary tables, figures and literature search strategy. [file 1471-2393-15-S2-S5-S2.docx]

Kangaroo mother care: a multi-country analysis of health system bottlenecks and solutions

Additional file 2

A. Table S1: Bottlenecks for kangaroo mother care 2

B. Table S2: Solutions for kangaroo mother care 12

C. Figure S1: Subnational grading of bottlenecks for kangaroo mother care 18

D. Literature search strategy 19

# A. Table S1: Bottlenecks for kangaroo mother care

| Healthy System Building Blocks | Category | Bottlenecks | Africa | | | | | | Asia | | | | | |
| --- | --- | --- | --- | --- | --- | --- | --- | --- | --- | --- | --- | --- | --- | --- |
|  |  |  | Cameroon | Democratic Republic of Congo (DRC) | Kenya | Malawi | Nigeria | Uganda | Afghanistan | Bangladesh | India | Nepal | Pakistan | Vietnam |
| Leadership and Governance  Leadership and Governance | **Policy/strategy/ guidelines** | Lack of policy for scale-up, regulatory bodies have not included as high impact intervention / promoted KMC |  |  | ✓ |  |  | ✓ |  | ✓ | ✓ |  | ✓ |  |
|  |  | KMC not part of national action plan |  |  |  |  |  |  |  |  |  |  |  | ✓ |
|  |  | Lack of institutionalizing KMC, project based |  |  |  |  |  | ✓ |  |  |  | ✓ | ✓ |  |
|  |  | No implementation and coordination mechanism between health and social sectors for supporting supervision and implementation of national guidelines on KMC |  |  |  |  |  |  |  |  |  |  |  | ✓ |
|  |  | Training materials available but no policy to implement them |  |  |  |  |  |  |  |  |  |  |  | ✓ |
|  |  | No maternity leave protection for majority of families |  |  |  |  |  | ✓ |  |  |  |  |  |  |
|  |  | No standards/guidelines for KMC in facilities even though recognized as priority intervention |  |  |  |  |  |  |  |  | ✓ | ✓ | ✓ | ✓ |
|  |  | KMC in guidelines but problems with dissemination and no guidance on setting up services (only paper, no practice) | ✓ |  |  |  | ✓ | ✓ |  |  |  |  |  |  |
|  | **Awareness** | No / limited awareness of KMC by leadership / concerned authorities |  |  |  |  |  |  |  |  | ✓ |  | ✓ | ✓ |
| Health Financing  Health Financing | **Policy** | No investment plan for scaling up / implementing KMC as part of sector programme / operational plan |  |  | ✓ |  |  |  |  | ✓ |  |  |  |  |
|  | **Funding** | Lack of funding for scale up and implementation (national or district level) |  |  |  |  |  |  | ✓ |  | ✓ | ✓ | ✓ | ✓ |
|  |  | Dependence on external funding |  | ✓ |  |  |  |  |  |  |  |  |  |  |
|  |  | Lack of budget in facilities to provide training for staff |  |  |  |  |  |  |  |  |  |  |  | ✓ |
|  | **Out-of-pocket expenditures** | Follow-up visits are expensive |  |  |  |  |  | ✓ |  |  |  |  |  |  |
|  |  | Costs of staying in hospital for family expensive – food and lodging (e.g. in Uganda leading to high demand for early discharge) | ✓ |  |  | ✓ | ✓ | ✓ |  |  | ✓ |  | ✓ |  |
| Health Workforce  Health Workforce | **Number, competence, distribution and type of health workers** | Shortage of competent health workers | ✓ | ✓ | ✓ |  |  |  |  | ✓ |  | ✓ | ✓ |  |
|  |  | Poor distribution of appropriately trained personnel |  |  |  |  | ✓ |  |  |  |  |  |  |  |
|  |  | No separate cadre available for KMC |  |  |  |  |  |  |  |  | ✓ |  |  |  |
|  | **Community health worker role** | ASHAs not empowered in KMC |  |  |  |  |  |  |  |  | ✓ |  |  |  |
|  | **Mentoring and supervision** | No mentoring guidelines and no routine supervision and mentoring |  |  |  |  |  | ✓ |  |  | ✓ | ✓ | ✓ |  |
|  |  | Few health workers have the support system to carry out KMC |  |  | ✓ |  |  |  |  |  |  |  |  |  |
|  | **Knowledge and awareness** | Poor awareness / knowledge of health workers in the importance of KMC in the care of LBW/preterm babies | ✓ |  |  |  | ✓ |  |  |  |  |  | ✓ | ✓ |
|  | **Health worker attitudes** | Poor health worker attitudes towards KMC (e.g. some health workers do not accept KMC as effective intervention, gender discrimination) |  |  |  |  | ✓ |  |  |  | ✓ |  | ✓ |  |
|  | **Training** | No KMC in pre-service/initial and overall training | ✓ |  | ✓ |  | ✓ | ✓ |  |  |  |  | ✓ | ✓ |
|  |  | No training curriculum |  |  |  |  |  |  |  | ✓ |  |  |  |  |
|  | **Job descriptions** | No job descriptions |  |  | ✓ |  |  |  |  |  | ✓ |  |  | ✓ |
| Essential Medical Products and Technologies | **Availability of supplies** | Lack of feeding and basic supplies | ✓ | ✓ |  | ✓ | ✓ |  |  | ✓ | ✓ |  | ✓ | ✓ |
|  |  | Lack of breast milk containers, pasteurizers for breast milk, refrigerators for milk storage |  |  | ✓ |  |  |  |  |  |  |  |  |  |
|  |  | Lack of cups |  |  |  |  |  |  |  |  |  |  | ✓ |  |
|  |  | Problems with electrical power and fridge and fuel costs |  | ✓ |  |  | ✓ |  |  |  |  |  |  |  |
|  |  | Pasteurizers for infants |  |  |  |  | ✓ |  |  |  |  |  |  |  |
|  | **Food for mother** | Lack of food for mothers performing KMC |  |  | ✓ |  |  |  |  |  |  |  |  |  |
|  | **Furniture** | Lack of beds, cupboards, room dividers |  |  |  |  |  | ✓ |  |  |  |  |  |  |
|  | **Procurement and supply chain** | Procurement and supply of basic equipment for KMC is not a priority for management - no vision |  |  |  |  |  |  |  |  |  | ✓ | ✓ |  |
| Health Service Delivery  Health Service Delivery | **Space and logistical constraints** | Facilities do not have adequate space for supporting and monitoring mothers and babies eligible for / practicing KMC (e.g. no separate ward / designated place for KMC and other logistical constraints though details not specified) | ✓ | ✓ | ✓ |  | ✓ |  |  | ✓ | ✓ | ✓ | ✓ |  |
|  | **Follow-up after discharge** | No sufficient follow-up mechanism or monitoring system for discharged babies receiving KMC |  |  |  |  | ✓ |  |  |  | ✓ | ✓ |  |  |
|  | **Service availability** | Referral advised for very LBW babies but then KMC not available in the referral facilities |  |  |  |  |  |  |  |  |  | ✓ |  |  |
|  | **Referral system (transport and access)** | Insufficient number of ambulances |  |  |  |  |  |  |  |  | ✓ |  |  |  |
|  |  | Poor referral and transport system |  |  |  |  |  |  |  |  | ✓ |  |  |  |
|  | **Quality** | No quality improvement and quality care programs to increase and improve implementation of KMC | ✓ |  |  |  | ✓ |  |  |  | ✓ | ✓ |  |  |
|  |  | KMC not included in clinical audits and perinatal death audits |  |  |  |  |  |  |  |  | ✓ |  |  |  |
|  | **Delivery** | KMC not part of service delivery – limited experience of working on this intervention |  |  |  |  |  |  |  |  |  |  |  | ✓ |
|  |  | Disparity in delivery of newborn services among localities and levels |  |  |  |  |  |  |  |  | O |  |  | ✓ |
| Health Information System  Health Information System | **Lack of information** | No KMC information, including coverage, captured in health information systems – new area with little or no expertise in some contexts | ✓ | ✓ | ✓ |  | ✓ | ✓ | ✓ | ✓ | ✓ | ✓ | ✓ | ✓ |
|  |  | No KMC information included in records | ✓ |  |  |  | ✓ |  |  |  |  |  | ✓ |  |
|  |  | KMC not mentioned in postnatal care/mother’s case sheets, discharge tickets, checklists for Auxiliary Nurse Midwives (ANMs) and Accredited Social Health Activists (ASHAs) and antenatal cards |  |  |  |  |  |  |  |  | ✓ |  | ✓ |  |
|  | **Quality of information** | Poor quality information on low birth weight/preterm babies |  |  |  |  |  | ✓ |  | ✓ |  |  |  |  |
| Community Ownership and Partnership  Community Ownership and Partnership  Community Ownership and Partnership | **Promotion** | No KMC in community and no strategy for promotion |  |  | ✓ |  |  |  |  |  |  |  | ✓ | ✓ |
|  |  | No Information Education & Communication in local language |  |  |  |  | ✓ |  |  |  | ✓ |  |  |  |
|  | **Financial barriers** | Financial barriers at community level |  |  |  |  |  | ✓ |  |  |  |  |  |  |
|  | **Awareness** | Lack of awareness and education, sensitization and mobilization in the community to increase knowledge on benefits of KMC | ✓ |  |  | ✓ | ✓ | ✓ | ✓ | ✓ | ✓ | ✓ |  | ✓ |
|  |  | Women have little knowledge regarding their rights |  |  |  |  |  |  |  |  | ✓ |  |  |  |
|  |  | New technique as far as this area is concerned |  |  |  |  |  |  |  |  |  |  | ✓ |  |
|  | **Access** | Large distances between community and health facilities |  |  |  |  | ✓ |  |  |  |  |  |  |  |
|  | **Acceptability** | Lack of comprehensive information on acceptability |  |  |  |  |  | ✓ |  |  |  |  |  |  |
|  |  | Not identified as an acceptable intervention in community – resistance to change | ✓ |  |  |  |  |  |  |  |  |  | ✓ |  |
|  | **Socio- cultural barriers** | Socio-cultural barriers (general) | ✓ |  |  |  |  |  |  |  | ✓ |  |  |  |
|  |  | Mothers used to carrying babies on back |  |  |  |  | ✓ |  |  |  |  |  |  |  |
|  |  | Milk banking not a concept that is well known / sharing another woman’s milk might be opposed based on traditional practices of marriage and inheritance |  |  |  |  | ✓ |  |  | ✓ |  |  |  |  |
|  |  | Perception that KMC is not feasible in hot, humid environment privacy will be a concern for implementing KMC |  |  |  |  |  |  |  | ✓ |  |  |  |  |
|  |  | Perception that privacy is a concern for implementing KMC |  |  |  |  |  |  |  | ✓ |  |  |  |  |
|  | **Misconceptions** | General misconceptions |  |  |  |  | ✓ |  |  |  |  |  |  |  |
|  |  | Myth that the most effective care is in an incubator | ✓ |  |  |  |  |  |  |  |  |  |  |  |
|  | **Engagement and support** | Poor involvement and support of men and community |  | ✓ |  |  | ✓ |  |  |  | ✓ |  |  |  |

# B. Table S2: Solutions for kangaroo mother care

| **Health system building block** | **Solutions** | **Africa** | | | | | | **Asia** | | | | | |
| --- | --- | --- | --- | --- | --- | --- | --- | --- | --- | --- | --- | --- | --- |
|  |  | **Cameroon (CAM)** | **Democratic Republic of Congo (DRC)** | **Kenya (KEN)** | **Malawi (MAL)** | **Nigeria (NGA)** | **Uganda (UGA0** | **Afghanistan (AFG)** | **Bangladesh (BGD)** | **India (IND)** | **Nepal (NPL)** | **Pakistan (PAK)** | **Vietnam (VTN)** |
| **Leadership and governance**  **Leadership and governance** | Advocacy and sensitisation – policy, awareness and increasing budget, necessary equipment/material, sensitise community and health workers, awareness on benefits of KMC to health workers and communities (DRC, KEN, BGD, VTN) |  | ✓ | ✓ |  |  |  |  | ✓ |  |  | ✓ | ✓ |
|  | Create policies (Nigeria - support passage and implementation of pending health bill) (KEN, NGA, IND) |  |  | ✓ |  | ✓ |  |  |  | ✓ |  |  |  |
|  | Include KMC in national MCH objectives (KEN, VTN) |  |  | ✓ |  |  |  |  |  |  |  |  | ✓ |
|  | Develop and disseminate guidelines - Include feeding practices for Low Birth Weight / preterm babies in national guidelines and milk banking (NGA, BGD, IND, VTN) |  |  |  |  | ✓ |  |  | ✓ | ✓ |  |  | ✓ |
|  | Develop implementation modalities at facilities and communities (BGD) |  |  |  |  |  |  |  | ✓ |  |  |  |  |
|  | Identify level of care where KMC should be implemented (IND) |  |  |  |  |  |  |  |  | ✓ |  |  |  |
|  | KMC in curriculum (IND) |  |  |  |  |  |  |  |  | ✓ |  |  |  |
|  | Strengthen Ministry of Health (PAK) |  |  |  |  |  |  |  |  |  |  | ✓ |  |
|  | Pilot before scale – positive practices (PAK) |  |  |  |  |  |  |  |  |  |  | ✓ |  |
| **Health Financing** | Advocacy for increased health budget line/allocation/funding for maternal and newborn health and evaluation (CAM, DRC, NGA, PAK, VTN) | ✓ | ✓ |  |  | ✓ |  |  |  |  |  | ✓ | ✓ |
|  | Develop a costed master plan to address the dissemination of policy and guidelines, space issues and socio-cultural practices (NGA) |  |  |  |  | ✓ |  |  |  |  |  |  |  |
|  | Increase donor support (BGD) |  |  |  |  |  |  |  | ✓ |  |  |  |  |
|  | Support establishment of community-based insurance schemes (NGA) |  |  |  |  | ✓ |  |  |  |  |  |  |  |
|  | Creation and expansion of mutual health (DRC) |  | ✓ |  |  |  |  |  |  |  |  |  |  |
| **Health workforce** | Training / capacity development of health workers (including pre-service and refresher training, KMC training in medical school and development of national training curriculum/guidelines to include all priority interventions) (CAM, DRC, KEN, NGA, BGD, IND, PAK, VTN) | ✓ | ✓ | ✓ |  | ✓ |  |  | ✓ | ✓ |  | ✓ | ✓ |
|  | Create dedicated cadre for KMC and increase number and capacity of existing health workers – including recruitment, retention and re-distribution (DRC, NGA, BGD, IND) |  | ✓ |  |  | ✓ |  |  | ✓ | ✓ |  |  |  |
|  | Job descriptions (KEN, IND) |  |  | ✓ |  |  |  |  |  | ✓ |  |  |  |
|  | Develop and implement mentoring guidelines (KEN, IND) |  |  | ✓ |  |  |  |  |  | ✓ |  |  |  |
|  | Set up monitoring, evaluation and supervision for KMC (IND, PAK, VTN) |  |  |  |  |  |  |  |  | ✓ |  | ✓ | ✓ |
| **Essential Medical Products and technologies**  **Essential Medical Products and technologies** | Ensure budget process includes funding for equipment (pasteurisers, refrigerators, etc.) that will promote adequate implementation of KMC (NGA) |  |  |  |  | ✓ |  |  |  |  |  |  |  |
|  | Advocate and establish procurement of basic supplies e.g. breast milk containers, refrigerators, clothing and food for mothers, weighing scale (KEN, BGD, IND) |  |  | ✓ |  |  |  |  | ✓ | ✓ |  |  |  |
|  | Develop standard list of equipment in facilities (IND, VTN) |  |  |  |  |  |  |  |  | ✓ |  |  | ✓ |
| **Health service delivery** | Invest in space for KMC – advocacy and policy (CAM, KEN, BGD, IND) | ✓ |  | ✓ |  |  |  |  | ✓ | ✓ |  |  |  |
|  | KMC follow-up can be part of existing services such as VHND services, HBPNC care of ASHA in India (IND) |  |  |  |  |  |  |  |  | ✓ |  |  |  |
|  | KMC established in Special Newborn Care Units (SNCUs) step down unit and Postnatal Care ward (IND) |  |  |  |  |  |  |  |  | ✓ |  |  |  |
|  | Integrate KMC and breastfeeding implementation into scoring system for evaluation of health facility performance (VTN) |  |  |  |  |  |  |  |  |  |  |  | ✓ |
| **Health information system**  **Health information system** | Develop indicators for KMC and incorporate into data collection tools (CAM, DRC, NGA, BGD, IND, VTN) | ✓ | ✓ |  |  | ✓ |  |  | ✓ | ✓ |  |  | ✓ |
|  | Include in records (CAM, IND) | ✓ |  |  |  |  |  |  |  | ✓ |  |  |  |
|  | Conduct clinical audits (IND) |  |  |  |  |  |  |  |  | ✓ |  |  |  |
| **Community ownership and partnership**  **Community ownership and partnership** | Better Information Education and Communication programs and empowering community health workers in KMC, improve community participation and engagement – community activities to include KMC and support groups and use of existing community structures (village health committees, community health committees, etc.) (CAM, MAL, NGA, BGD, IND) | ✓ |  |  | ✓ | ✓ |  |  | ✓ | ✓ |  |  |  |
|  | Ensure implementation of community outreach services by community health workers that are appropriately supervised and monitored and use a platform for dissemination of essential health information (NGA) |  |  |  |  | ✓ |  |  |  |  |  |  |  |
|  | Create effective action plan to address community perceptions (PAK) |  |  |  |  |  |  |  |  |  |  | ✓ |  |
|  | Translation of materials in local languages (KEN) |  | ✓ |  |  |  |  |  |  |  |  |  |  |
|  | Increase awareness of male involvement (KEN) |  | ✓ |  |  |  |  |  |  |  |  |  |  |
|  | Popularise KMC in facilities (CAM) | ✓ |  |  |  |  |  |  |  |  |  |  |  |

# C. Figure S1: Subnational grading of bottlenecks for kangaroo mother care


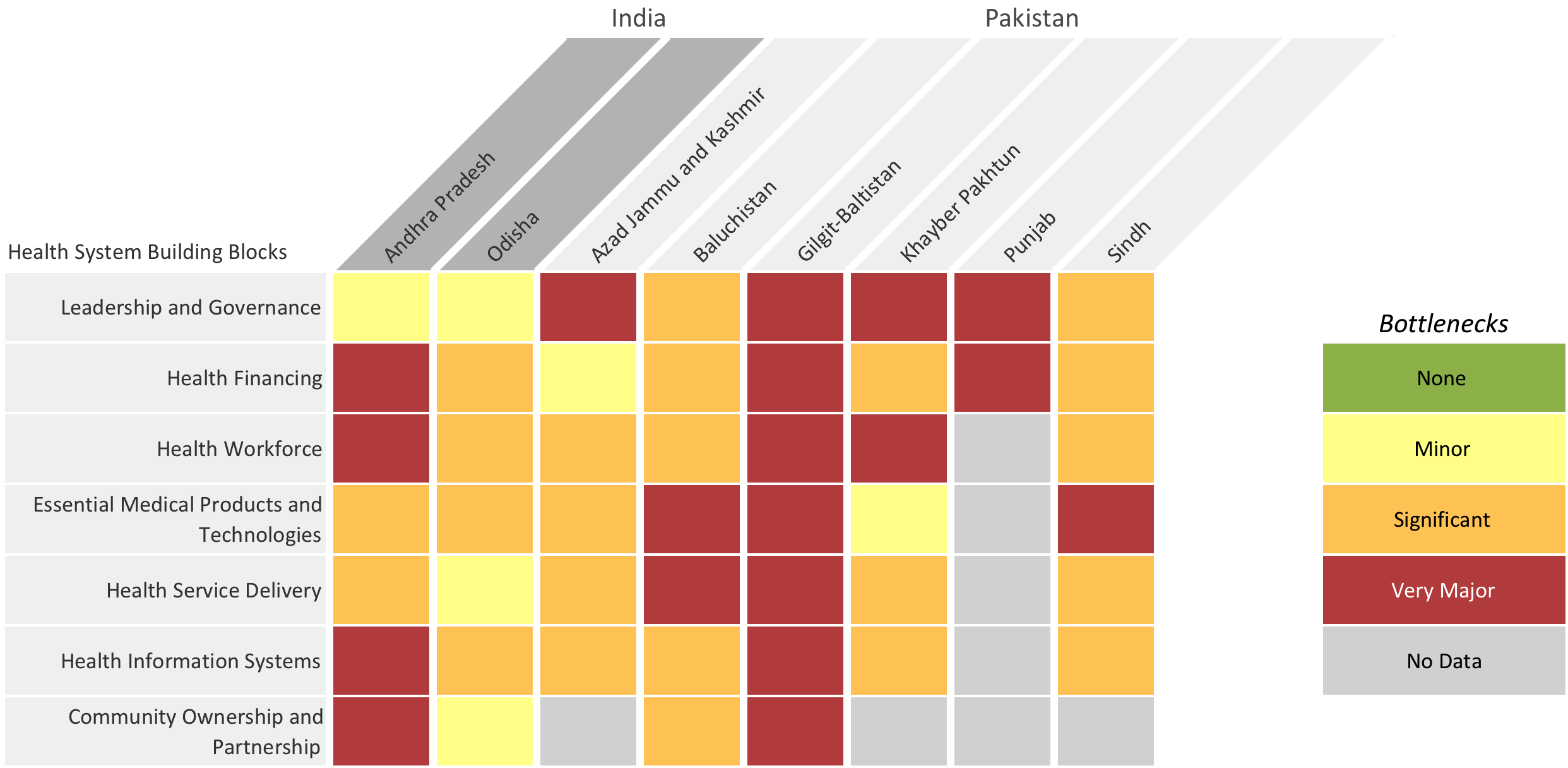


# D. Literature search strategy

**For the background section, we used the following search terms in Pub Med. Limits were applied and only relevant articles were retrieved. We also looked at citations of identified articles to find other relevant articles.**

1. (kangaroo mother care OR KMC OR skin-to-skin OR skin to skin) AND (implement* OR scale-up OR scale up)
2. (bottleneck OR challenge OR barrier OR obstacle) AND (newborn OR neonate OR baby OR babies OR infant) AND (kangaroo mother care OR KMC OR skin-to-skin care OR skin to skin)

**For the discussion section we searched the following terms in pub med and google. Only relevant articles were retrieved.**

**Leadership and governance**

(Health) AND (Leadership OR governance OR government OR ownership OR regulat* OR guideline OR strateg* OR policy) AND (barrier OR bottleneck OR obstacle OR challenge)

Implementation models/modalities

KMC policies and guidelines

**Community:**

(Health) AND (barrier OR bottleneck OR obstacle OR challenge) AND (information education communication OR community mobilization OR community OR awareness OR ownership OR empowerment OR sensitization)

Community awareness

Community and facility linkages

Community engagement

**Health Financing**

(Health) AND (barrier OR bottleneck OR obstacle OR challenge) AND (financial access OR financial barrier OR out-of-pocket payment OR user fees OR funding OR budget OR donor OR insurance)

Budget allocation

Innovative funding

Mutual health insurance schemes

National health insurance

**Health workforce**

(Heath worker OR staff OR health provider OR human resource) AND (barrier OR bottleneck OR obstacle OR challenge) AND (pre-service training OR in-service training OR task-shifting OR training OR retention OR distribution OR supervision)

Community health workers

Task-shifting

**Health service delivery**

(Heath worker OR staff OR health provider OR human resource OR health delivery OR health service delivery OR provid* care) AND (barrier OR bottleneck OR obstacle OR challenge) AND (space OR facility OR health centre OR NICU OR referral OR follow-up)

Referral mechanism

Care for preterm/LBW babies
